# Apolipoprotein E COG 133 mimetic peptide improves 5-fluorouracil-induced intestinal mucositis

**DOI:** 10.1186/1471-230X-12-35

**Published:** 2012-07-13

**Authors:** Orleâncio Gomes R Azevedo, Renato André C Oliveira, Bruna Castro Oliveira, Snjezana Zaja-Milatovic, Celina Viana Araújo, Deysi Viviana T Wong, Tiê Bezerra Costa, Herene Barros Miranda Lucena, Roberto César P Lima-Júnior, Ronaldo A Ribeiro, Cirle A Warren, Aldo Ângelo M Lima, Michael P Vitek, Richard L Guerrant, Reinaldo B Oriá

**Affiliations:** 1 Center for Global Health, School of Medicine, University of Virginia, Carter Harrison Bldg MR-6, 625 Crispell Drive, Room 2526, Charlottesville, VA, 22908, USA; 2 Laboratoy of the Biology of Tissue Healing, Ontogeny and Nutrition, Institute of the Brazilian Semi-arid, School of Medicine, Federal University of Ceara, Rua Coronel Nunes de Melo, 1315 Rodolfo Teófilo, Fortaleza, Ceará, 60.430-270, Brazil; 3 Cognosci Inc., Duke University, Research Triangle Park, Durham, NC, USA; 4 Laboratory of Inflammation and Cancer, School of Medicine, Federal University of Ceara, Rua Coronel Nunes de Melo, 1315 Rodolfo Teófilo, Fortaleza, Ceará, 60.430-270, Brazil

**Keywords:** Mucositis, Apolipoprotein E, 5-fluorouracil, Inflammation, Cytokines

## Abstract

**Background:**

Intestinal mucositis is one of the major troublesome side effects of anticancer chemotherapy leading to poor patient compliance. In this study we addressed the role of the novel apolipoprotein E (ApoE) COG 133 mimetic peptide in 5-fluorouracil (5-FU)-challenged Swiss mice and IEC-6 cell monolayers. Experiments were also conducted in C57BL6J ApoE knock-out mice to assess the effects of apoE peptide treatment.

**Methods:**

Experimental groups were as follows: unchallenged controls, 5-FU-challenged mice (450 mg/kg, i.p) with or without the ApoE peptide (0.3, 1, and 3 μM, given twice daily i.p. for 4 days). Mice were sacrificed 3 days after 5-FU challenge. Proximal small intestinal samples were harvested for molecular biology and histological processing. We conducted ELISA assays and RT-PCR to target IL-1β, TNF-α, IL-10, iNOS, and myeloperoxidase (MPO) to assess intestinal inflammation. Cell death and NF-κB assays were also conducted in apoE knock-out mice. In our in vitro models, IEC-6 cells were exposed to 1 mM of 5-FU in glutamine free media with or without the ApoE peptide (0.02, 0.2, 2, 5, 10, and 20 μM). We investigated IEC-6 cell proliferation and migration, 24 h after the 5-FU challenge. Additionally, apoptotic IEC-6 cells were measured by Tunel and flow cytometry. Equimolar doses of the ApoA-I (D4-F) peptide were also used in some experiments for comparative studies.

**Results:**

Villus blunting and heavy inflammatory infiltrates were seen in the 5-FU-challenged group, findings that were partially ameliorated by the ApoE peptide. We found increased intestinal MPO and pro-inflammatory IL-1β and TNF-α levels, and TNF-α and iNOS transcripts, and reduction of IL-10 following 5-FU treatment, each of which were partially abrogated by the peptide. Improvements were also found in IEC-6 cell apoptosis and migration following ApoE and D-4F treatment.

**Conclusion:**

Altogether, these findings suggest that the novel ApoE COG 133 mimetic peptide can reduce 5-FU-induced intestinal changes and potentially benefit mucositis.

## Background

The intestinal barrier function is very susceptible to injury caused by anticancer drugs due to its rapid epithelial turnover rate of about 3–4 days [1]. Mucositis is a side effect of chemotherapy in which there is a dramatic drop in quality of life of patients, sometimes leading to discontinuation of therapy. Chemotherapy cytotoxic effects vary in severity according to therapy schemes and patient susceptibility, many of them may lead to intestinal mucosal damage, heavy inflammatory responses, often associated with increased epithelial cell death, intestinal epithelial breakdown, and luminal bacterial translocation [2,3].

5-Fluorouracil (5-FU) is widely used for colorectal cancer [4], but enteric inflammation accompanied by diarrhea is a commonly reported side-effect. Intestinal mucositis with diarrhea and vomiting is often the dose-limiting adverse effect of 5-FU therapy and is likely accompanied by poor treatment compliance.

Previous studies have demonstrated that 5-FU-treated rats show significant reductions in villus height in the upper small bowel, with increased epithelial cell damage, increased leukocyte infiltrates and pro-inflammatory cytokines in the intestinal mucosa [5]. In addition, our previous work found beneficial effects of alanyl-glutamine and glutamine in reducing mucositis and accelerating mucosal healing, following 5-FU challenge in Swiss mice [6]. In vitro studies with IEC-6 monolayers exposed to 5-FU and under glutamine supplementation also confirmed a pro-mitotic activity of these gut-trophic nutrients [7].

Recently, a novel apolipoprotein E (ApoE) COG 133 mimetic peptide, comprising residues 133–149 of the human apolipoprotein E has been reported [8]. This mimetic peptide competes with the ApoE holoprotein for binding the LDL receptor, with potent anti-inflammatory properties in models of brain injury, yet preserving the neuroprotective role of the holoprotein [9,10].

Investigators have found consistent anti-inflammatory actions of a variety of ApoE mimetic peptides, with reductions in several pro-inflammatory cytokines and less activation of NF-κB [8,11]. In addition, ApoE mimetic peptides have been found to inhibit NF-κB signaling in a model of colitis supporting a role of this peptide in the intestinal tract [12]. In the present study we investigated whether an anti-inflammatory role of the ApoE COG 133 peptide could improve the 5-FU-induced epithelial damage assessed in Swiss mice and whether it could enhance epithelial healing using *in vitro* monolayers of undifferentiated crypt-derived IEC-6 cells. In addition, to assess whether the ApoE COG 133 peptide could reverse the mucosal damage-induced by the 5-FU challenge in the absence of the endogenous protein, we also conducted experiments with ApoE knock-out mice and their wild-type controls. To our knowledge this is the first study addressing the role of the apopoliprotein E in 5-FU-induced intestinal mucositis.

## Methods

### In vivo studies

#### Drugs

5-fluorouracil (Eurofarma, SP, Brazil) was obtained from the Laboratory of Inflammation and Cancer (LAFICA)/Federal University of Ceara for the intestinal mucositis induction. The ApoE COG133 and apoA-1 (D-4F) peptide were provided by Dr. Michael Vitek at Duke University (Durham, North Carolina, USA). The ApoE COG 133 and apoA-I (D-4F) peptides were synthesized from the Peptide Synthesis Laboratory at the University of North Carolina (Chapel Hill, NC) to a purity of 95% and reconstituted in sterile isotonic PBS. The ApoE COG 133 amino terminus was acetylated, and the carboxyl terminus was blocked with an amide moiety. The 17-amino acid peptide was derived from ApoE residues 133–149 (the receptor binding region): Ac-LRVRLASHLRKLRKRLL-amide. The lyophilized peptides (ApoE COG 133 and D-4F) were diluted in distilled water and saved frozen as stock solution and aliquot to prepare experimental doses.

#### Animals

Swiss and C57BL6J ApoE wild-type and knock-out mice (weighing between 25 and 30 g) were obtained from the Department of Physiology and Pharmacology vivarium, Federal University of Ceará. All experimental protocols were in compliance with the Brazilian College for Animal Experimentation (COBEA) and the Animal Care and Use Committee guidelines from the Federal University of Ceará. Experimental mice were kept in polyethylene boxes with free access to chow diet and water until sacrifice, and subjected to 12 h light–dark cycles.

#### Mucositis induction, ApoE treatment and tissue collection

Intestinal mucositis was induced by single 5-fluorouracil administration (450 mg/kg) intraperitoneally (i.p) in Swiss and ApoE wild-type and knock-out mice, which was considered as day 1st. ApoE COG 133 peptide was given daily (12/12 h, i.p. 0.3, 1.0 and 3.0 μM) during the 1st to 4th days following the 5-FU challenge. Animals were weighed daily. The euthanasia was performed on the 4th day in anesthetized mice with an overdose of ketamine/xylazine solution. After sacrifice, 1 cm-long segments of the proximal small intestine were harvested and immediately frozen in liquid nitrogen and thereafter stored in −80°C freezer until use. Each first 1-cm long segment after the pyloric valve was picked and stored in buffered formaldehyde solution for further histology processing.

#### Leukocyte counts

In order to evaluate the 5-FU myelocytotoxicity, leukocyte counting was performed using Newbauer chambers. Swiss mice were slightly anesthetized with ether, and a capillary tube was inserted into the orbital plexus to draw peripheral blood, which was mixed with a 3% acetic acid solution (Turk’s solution) at a 1:20 ratio for loading. Final counts were given as the number of cells per mm^3^.

#### Villus height and crypt mitotic index

In order to evaluate the role of ApoE COG 133 in healing the injured intestinal mucosa, we measured villus height in at least 10 villus longitudinal sections using low-magnified H&E-stained photomicrographs by Image J software 1.4 ® (NIH - National Institutes of Health, Bethesda, MD, USA) after proper calibration. Villus height was measured from the base-apex longitudinal axis. In order to find whether the ApoE COG 133 peptide restores epithelial cell renewal in ApoE knock-out mice, the mitotic index was also calculated in these mouse strains by blindly counting well-defined mitotic figures at the crypt proliferative compartment, as described elsewhere [6]. Measures were done under light microscopy using an immersion objective (1000X). The absolute values were averaged to produce the mitotic index of each group.

#### Inflammation markers

##### Myeloperoxidase (MPO) assay

MPO tissue activity is used as a marker of inflammatory cell infiltrates (especially neutrophils) in the inflamed tissue [13]. Segments from the proximal small intestine were incubated in 0.5% HTAB (hexadecyltrimethylammonium bromide) solution at a ratio of 50 mg of tissue/ml of buffer, and homogenized and centrifuged (1500 *G*/15 min at 4°C). The supernatant was transferred to an Epperdorf tube and further centrifuged (10 min). After plating of the supernatant (7μL onto a 96- plate well), 200 μL of the reading solution (5 mg of O-dianisidine, 15 μl of 1% H_2_O_2_, 3 ml of phosphate buffer and 27 ml of H_2_O) were added and read at 450 nm (t_0_ = 0 min and t_1_ = 1 min). The change in absorbance was obtained and expressed as MPO/mg tissue (MPO activity).

#### ELISA cytokine assay

Specimens were stored at −80°C until required for assay. The tissue collected was homogenized and processed as described by Soares et al [14]. The detection of TNF-α, IL-1β, and IL-10 concentrations was determined by ELISA. Briefly, microtiter plates were coated overnight at 4°C with antibody against murine TNF-α, IL-1β, and IL-10 (2 μg/ml). After blocking the plates, the samples and standard at various dilutions were added in duplicate and incubated at 4°C for 2 h. The plates were washed three times with buffer. After washing the plates, biotinylated sheep polyclonal anti-TNF-α or anti-IL-1β or anti-IL-10 (diluted 1:1000 with assay buffer 1% BSA) was added to the wells. After further incubation at room temperature for 1 h, the plates were washed and 50 μl of avidin-HRP diluted 1:5000 were added. The color reagent o-phenylenediamine (OPD; 50 μl) was added 15 min later and the plates were incubated in the dark at 37°C for 15–20 min. The enzyme reaction was stopped with 2 N H_2_SO_4_ and absorbance was measured at 490 nm. Values were expressed as picograms/milliliter (pg/ml).

#### Reverse transcriptase-PCR

Based on our previous results, described above, we chose the 3 μM, as the best ApoE mimetic peptide dose for the RT-PCR studies. Briefly, proximal intestinal samples were immediately immersed and frozen in liquid nitrogen and stored at −80°C until immediately before analyses. Samples were thawed and resuspended in a denaturating solution (4 M guanidinium thiocyanate, 25 mM sodium citrate, pH 7, 0.5% sarcosyl) and extracted using RNeasy mini columns, according to manufacturer’s instructions (RNeasy mini kit, Qiagen, Valencia, California). Total RNA was quantified and checked for purity (A260:280 ratio) by standard spectrophotometry (Biophotometer, Eppendorf, Hamburg, Germany). First strand of cDNA was synthesized from 2 μg of total RNA using oligo(dT)12-18 as primers in the presence of Moloney murine leukemia virus reverse transcriptase (Superscript™ Reverse Transcriptase, Invitrogen, Carlsbad, California), for 1 hr at 37 C. PCR was carried out with 5 μl of the RT-product (50 μl final reaction volume) with 5 μl of reverse and forward murine primers (Invitrogen) for TNF-α and iNOS. Primers were according to Coutinho et al, 2010 [15].

Amplicons were generated in thermal cycler (Biorad, Hercules, CA) conditions. The temperature profile of the amplification consisted of 35 cycles of 45 s denaturation at 95 C and 2.5 min annealing and extension at 60 C. Negative controls were without RNA. PCR products were separated onto 2% agarose gel and visualized by ethidium bromide staining and photographed digitally using a gel documentation system (Alpha Imager, Alpha Innotech Corp., San Leandro, California). The housekeeping gene, β-actin, was used as an internal control. Sample band densities were ratio-normalized by using β-actin band intensities, obtained within the same PCR reaction, with the aid of NIH Image J software (NIH, Bethesda, Maryland).

#### NF-κB immunohistochemistry

Immunohistochemistry for NF-κB p50 (NLS) was performed on proximal intestine tissue from ApoE knock-out mice and wild-type controls using the streptavidin-biotin-peroxidase method in formalin-fixed, paraffin-embedded tissue sections (4μm thick), mounted on poly-L-lysine-coated microscope slides. The sections were deparaffinized and rehydrated through xylene and graded alcohols. After antigen retrieval, endogenous peroxidase was blocked (15 min) with 3% (vv-1) hydrogen peroxide and washed in phosphate-buffered saline (PBS). Sections were incubated overnight (4°C) with primary rabbit anti-NF-κB antibody diluted 1:200 in PBS plus bovine serum albumin (PBS-BSA). The slides were then incubated with biotinylated goat anti-rabbit; diluted 1:400 in PBS-BSA. After washing, the slides were incubated with avidin-biotin-horseradish peroxidase conjugate (Strep ABC complex by Vectastain® ABC Reagent and peroxidase substrate solution) for 30 min, according to the Vectastain protocol. NF-κB was visualized with the chromogen 3,3′diaminobenzidine (DAB). Negative control sections were processed simultaneously as described above but with the first antibody being replaced by PBS-BSA 5%. None of the negative controls showed NF-κB immunoreactivity. Slides were counterstained with Harry’s hematoxylin, dehydrated in a graded alcohol series, cleared in xylene and coverslipped.

#### Apoptotic assay

In order to find whether the ApoE COG 133 peptide could restore cell viability in ApoE knock-out mice, we also performed a Tunel assay. The fragmentation of DNA was examined using a commercial kit for the detection of apoptosis (ApopTag® Plus Flourescein in situ Apoptosis Dectection kit, Chemicon, Temecula, CA, USA) using the Tunel technique. After deparaffinization and hydration, sections were subjected to enzymatic digestion with 20 μg/mL of proteinase K for 15 min, washed with phosphate-buffered saline (PBS), 0.1 M, pH 7.4. They were then immersed in an equilibration buffer for 10 min and incubated with a stock solution of terminal deoxynucleotidyl transferase enzyme at 37°C for 1 h. They were then washed with PBS and incubated with antidigoxygenin-fluorescein conjugate for 30 min in the dark using a humidified chamber. Apoptotic figures were then visualized using a confocal laser scanning microscope (FV100, Olympus, USA). Tunel-labeled cells were counted and averaged as total labeled cells per high magnification field, according to experimental groups.

### In vitro studies

#### Cell culture

Rat crypt jejunal cells (IEC-6) were cultured at 37°C in a 5% CO_2_ incubator. The maintenance cell media used was Dulbecco’s Modified Eagle Media (DMEM; Gibco BRL, Grand Island, NY) supplemented with 5% fetal calf serum (FCS), 5 mg bovine insulin, 50 μg/ml of penicillin/streptomycin (DMEM; Gibco BRL, Grand Island, NY) and a final concentration of 1 mM of sodium pyruvate. The media were changed thrice a week, according to standard culture protocols. Dulbecco’s Modified Eagle Medium without Gln (DMEM; Gibco BRL) was used whenever the supplementation effect of the ApoE novel peptide was evaluated. We tested the effect of the COG133 peptide (ApoE 133) and D-4F peptide (ApoA-I) at 0.02, 0.2, 2, 5, 10, and 20 μg/ml. The D4-F peptide was chosen since it has been found as anti-inflammatory action in models of cardiovascular disease [16]. Both peptides were supplied as a lyophilized powder and reconstituted in sterile water and aliquot into smaller sample sizes for dosing experiments in sterile lactated ringers stored at −80°C.

#### Wound Healing Assay

For wounding assay, we used rat jejunal epithelial cells, IEC-6 cells (passage 19). IEC-6 cells were seeded in 6 well plates at a concentration of 10^6^ cells per well and were allowed to grow until full confluence, before wounding cells were challenged with 1 mM of 5-FU. After 1 hour of treatment with 5-FU, cells were washed twice with 1xPBS and scratched with a sterile razor blade at the midline extending to the right side of the well. The intention of the scrape is to simulate epithelial damage and measure cell migration from the injured site during the early phase of tissue recovery. After wounding, the wells were washed again, two times with 1xPBS and the media were changed to glutamine-free media containing different concentrations (0.02; 0.2; 2.0; 5.0; 10, and 20. μM) of mimetic peptides ApoE COG133 and D4F. Migrated cells were observed under 10X magnification after 24 h using Olympus Inverted microscope with QImaging camera. Areas of the farthest cell migration were chosen, and images were taken using QCapture Pro.5.1.software. Cells were manually counted and expressed as number of cells per mm^2^.

#### Cell proliferation assays

##### WST assay

Cell proliferation was measured indirectly using the tetrazolium salt WST-1 (4-[3-(4-iodophenyl)-2 H-5-tetrazolio]-1-3-benzene disulfonate), according to the manufacturer’s recommendations. A 96-well plate was seeded with IEC-6 cells in a total concentration of 4 x 10^4^ cells/mL in 100μL of standard DMEM media. Cells were allowed to attach for 48 h, when the media volume was removed and changed to standard medium (SM) (0.01–100nM) or not, and then either incubated or not with 5-FU (1 mM) for 1 hour. After 24 h and 48 h, wells were incubated for 2 h with 10 mL of the tetrazolium salt and the absorbance was measured using an ELISA microplate reader at 450 nm (reference range 420–480 nm). Tetrazolium salts are cleaved to formazan by mitochondrial enzymes in viable cells. Enhancement of the number of viable cells will result in an increase of the amount of the formazan dye, which is detectable by the ELISA reader. Therefore, this model indirectly measures the cell proliferation rate in a time dependent manner.

#### Ki67 Immunohistochemistry

IEC-6 cells were plated in 2-well tissue culture chamber slides at a concentration of 10^5^ cells per well. Cells were allowed to attach the chamber slide for 24 hours. After 24 hours, cells were challenged for 1 hour with 5-FU (1 mM), then washed twice with 1xPBS and treated with *either* ApoE COG133 or apoA-I D4F mimetic peptides in two different concentrations: 2. and 20μM. Cells were then exposed to mimetic peptides for 6 hours in glutamine-free medium. After the treatment cells were washed trice with 1xPBS and fixed in 4% paraformaldehyde (methanol free) for 15 minutes, then washed trice with 1xPBS. IEC-6 cells were stained by using Ki67 antibody (MKI67rabbit monoclonal primary antibody) from Epitomics Inc. (Burlingame,CA). Ki67 is a nuclear protein that is tightly linked to the cell cycle and lables cell proliferation. Working dilution of primary antibody was 1:400. Secondary antibody used was anti-rabbit from DAKO, (Carpinteria, CA). Staining was done in Tissue Research Core Facility at University of Virginia Medical School. Images were taken in the Core Facility using Olympus DP71 microscope and Microsuite Pathology Edition software using ×20 magnifications with a 100 μm scale. We evaluated 6 to 8 randomly selected areas. Ki67 positive cells were counted manually and calculated per total number of cells, expressed as percentage of Ki67 positive cells.

#### Cell death assays

##### Tunnel

IEC-6 cell were plated in 2-well tissue culture chamber slides at a concentration of 10^5^ cells per well. Cells were allowed to attach the chamberslide for 24 hours. After 24 hours cells were challenged for 1 hour with 1 mM 5-FU, then washed twice with 1 × PBS and treated with APOE COG133 and APOA-I D4F mimetic peptides in 6 different concentrations: 0.02; 0.2; 2.0; 5.0; 10, and 20μM. Cells were then exposed to mimetic peptides for 24 hours in glutamine-free medium. After the treatment cells were washed trice with 1 × PBS and fixed in 4% paraformaldehyde (methanol free) for 15 minutes, then washed trice with 1 × PBS. Cells were stained with using DeadEnd Fluorometric Tunel kit from (Promega*,* Madison, WI). Tunel System measures the fragmented DNA of apoptotic cells by catalytically incorporating fluorescein-12-dUTP at 3′-OH DNA ends using the enzyme TdT (Terminal deoxynucleotidyl Transferase). Apoptotic cells were visualized using DAPI (SouthernBiotech., Birmingham, AL). Images were taken under 10× and ×40 magnification using Fluorescent Olympus 1 × 71 Inverted microscope with QImaging camera, with QCapture Pro.5.1.software. We evaluated 10 randomly selected fields. Cells were manually counted per selected area and expressed in number of cells per mm^2^.

#### Flow cytometry

Apoptosis and necrosis were measured using the ApoAlert Annexin V kit. Annexin V is a molecule that binds to phosphatidylserine (PS) and, when conjugated to a fluorochrome, detects apoptotic cells expressing PS on the reversed membrane surface. For this protocol, propidium iodide (PI) was also used to detect necrotic and late apoptotic cells, which express PI inside the membrane. The cells were seeded on 12-well plates at a concentration of 5 × 10^5^ cells/well. These cells were allowed to attach to the plate surface for 24 h. Afterwards, cells were washed with DMEM Gln-free media and either incubated or not with 5-fluorouracil for 1 h. Wells were then washed again and supplemented with COG133 and then incubated for 24 h. Cells were trypsinized, centrifuged, and washed with serum containing media, before incubation with Annexin V. Cells were counted and diluted to 10^5^–10^6^ cells and were rinsed and resuspended in 200 μl of binding buffer. Five microliters of Annexin V and 10 μl of PI were added and incubated for 5–15 min in the dark. The samples were then processed at the University of Virginia’s Flow Cytometry Core, using a Calibur (Becton Dickinson) dual laser fluorescence-activated cell sorter (FACS). Low-concentrated bovine fetal serum was used to further evaluate peptide treatment under deprived media.

### Statistical analysis

The results are shown as mean ± SEM. Either analysis of variance ANOVA followed by Student Newman-Keuls for multiple group comparisons or Student’s *T* test for unpaired comparisons between two groups were used. *P* <0.05 was set to indicate significant differences.

## Results

### In vivo studies

#### Weight gain and leukocyte counts

As expected, 5-FU-injected mice (450 mg/Kg, i.p) (day of injection = day 1) show profound weight loss compared with the unchallenged controls from the 3rd day post-challenge (two days after 5-FU injection) (p < 0.001). ApoE COG 133 mimetic peptide treatment (0.3, 1 and 3 μM) did not improve weight loss caused by the high 5-FU dose used in our protocols in either Swiss mice or ApoE knock-out mice. 5-FU-challenged mice exhibited marked reductions (77.1%) in the leukocyte count (cell number/mm^3^ from peripheral blood), compared to the unchallenged control group (p < 0.001), confirming that the 5-FU-tested dose was cytotoxic to the bone marrow, and this effect was also not diminished by the ApoE COG 133 peptide administration (p > 0.05, by ANOVA), although a trend of increased leukocyte counts was seen. Nevertheless, when a group-by-group comparison between the 5-FU controls and the highest doses of the peptide was made, a significant improvement in the mean peripheral leukocyte number was found (p < 0.05, by unpaired Student *T* test) (data not shown).

#### Histology

**Figure 1:**
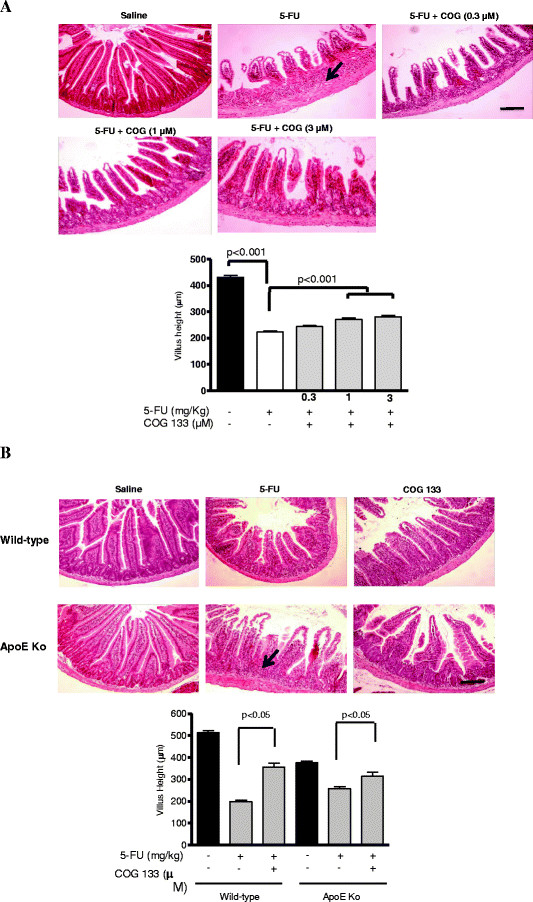
**A. Representative histology and villus height from H-E stained-proximal intestine (X100) of Swiss mice challenged by 5-FU-induced intestinal mucositis (450 mg/kg via i.p., single dose, and treated with the apoE mimetic peptide COG 133 (0.3, 1, and 3 μM, via i.p., 12/12 h, vol. 10 μl/g) or 0.9% saline (i.p.).** Scale bar = 100 μm. B. Representative histology and villus height from H-E stained-proximal intestine (X100) of C57BL6J ApoE knock-out and wild-type mice challenged by 5-FU-induced intestinal mucositis (450 mg/kg via i.p., single dose) and treated with the apoE mimetic peptide COG 133 (3 μM, via i.p., 12/12 h, vol. 10 μl/g) or 0.9% saline (i.p.). Scale bar = 100 μm. The arrow indicates a heavy inflammatory cell infiltrate in the submucosal tissue from the 5-FU-treated mice. Note reductions of inflamed tissue in apoE COG 133-treated groups, especially in the highest doses. Values represent mean ± SEM and were analyzed by one-way ANOVA and Bonferroni. At least 10 villi per animal were analyzed (n = 4 animals/group). Experimental mice were sacrificed on the fourth day post-challenge.

In our protocol, the 5-FU-induced intestinal mucositis features a heavy inflammatory response, with increased inflammatory cell infiltrates in the submucosa and mucosa, mostly invading polymorphonuclear leukocytes. In addition, profound villous atrophy, with villus blunting and crypt derangement are seen, as opposed to the unchallenged control (Figure 1). The ApoE mimetic peptide improved intestinal inflammation cell infiltrates and villus height (doses of 1 and 3 μM, p <0.001), as compared with the untreated challenge mice (Figure 1A). Since, the dose of 3 μM was beneficial to 5-FU-treated Swiss mice, we also tested this dose in the experiments conducted with the ApoE knock-out mice. ApoE COG mimetic peptide (3 μM) was also able to improve the duodenal histology following 5-FU treatment in ApoE deficient and wild-type mice, with significant improvements in villus height (Figure 1B).

**Figure 2:**
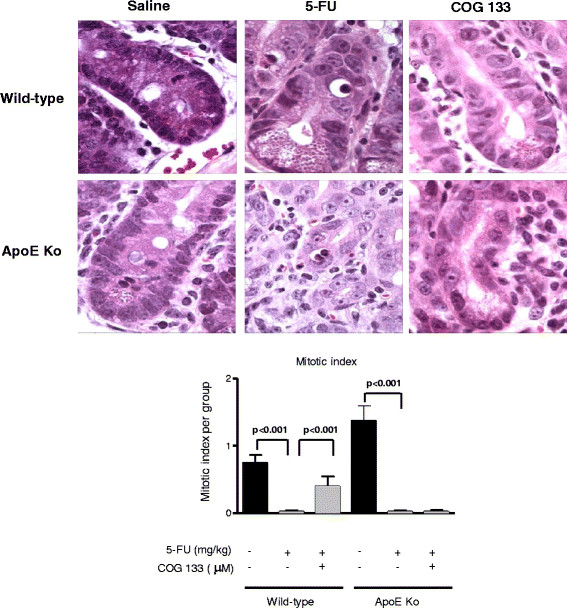
**Mean mitotic index of the H&E stained-duodenal crypts (X1000) from C57BL6J ApoE knock-out and wild-type mice challenged by 5-FU-induced intestinal mucositis (450 mg/kg via i.p., single dose) and treated with the apoE mimetic peptide COG 133 (3 μM, via i.p., 12/12 h, vol. 10 μl/g) or 0.9% saline (i.p.).** Values represent mean ± SEM and were analyzed by one-way ANOVA and Bonferroni. At least 10 crypts per animal were analyzed (n = 4 animals/group).Experimental mice were sacrificed on the fourth day post-challenge.

5-FU-treated mice show profound cell proliferation impairment, as demonstrated by ablated mitotic index in the duodenal crypts. The ApoE COG 133 peptide at 3 μM significantly increased the mitotic crypt numbers in C57BL6J wild-type animals but not in ApoE knock-out mice. In addition, COG 133 treatment improved crypt architecture and reduced lamina propria inflammation, compared to untreated controls (Figure 2).

#### Myeloperoxidase (MPO) and ELISA cytokine assays

**Figure 3:**
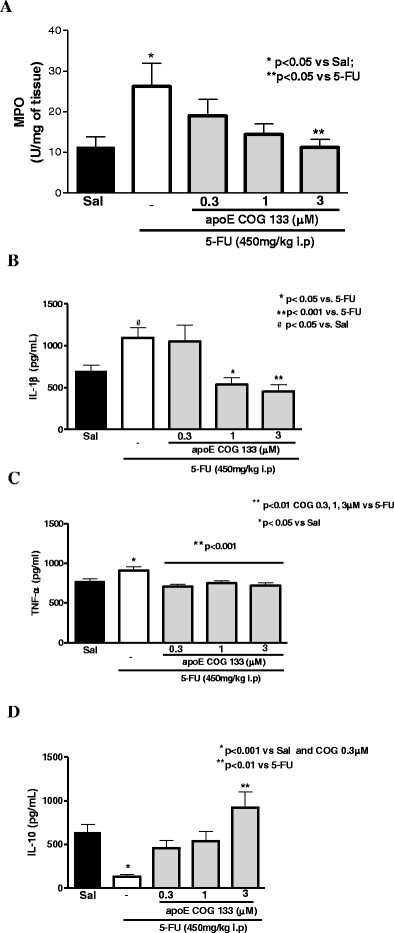
**A. Proximal intestinal myeloperoxidase (MPO) assay from Swiss mice challenged by 5-FU-induced intestinal mucositis (450 mg/kg via i.p., single dose) and treated with the apoE mimetic peptide COG 133 (0.3, 1, and 3 μM, via i.p., 12/12 h, vol. 10 μl/g) or 0.9% saline (i.p.). B, C, and D.** IL-1β, TNF-α, and IL-10 Elisa assays, respectively, from Swiss mice challenged by 5-FU-induced intestinal mucositis (450 mg/kg via i.p., single dose) and treated with the apoE mimetic peptide COG 133 (0.3, 1 and 3 μM, via i.p., 12/12 h, vol. 10 μl/g) or 0.9% saline (i.p.). Proximal intestine segments were harvested from experimental mice on the fourth day. Values represent mean ± SEM and were analyzed by one-way ANOVA. At least four animals were used per group. At least N = 7 for all groups.

We found increased intestinal myeloperoxidase levels (an enzyme found in neutrophil azurophilic granules), following 5-FU administration. ApoE COG 133 peptide caused a significant reduction in the intestinal MPO levels at the maximum dose tested (3 μM) (p <0.05) (Figure 3A). Additionally, we found increased intestinal IL-1β levels following 5-FU treatment, an effect that was partially abrogated by the ApoE peptide administration at doses of 1 and 3 μM (p < 0.05 and p < 0.001, respectively), Figure 3B.

As seen in Figure 3**C,** we also found increased TNF-α levels in the proximal small intestine from 5-FU-treated mice, which were partially decreased after ApoE COG 133 peptide administration in all doses tested (p <0.001). On the other hand, 5-FU administration caused markedly reduced intestinal IL-10 levels, effects that were again improved with ApoE peptide treatment (at highest dose) (p < 0.001) (Figure 3D).

#### Reverse Transcriptase-PCR

**Figure 4:**
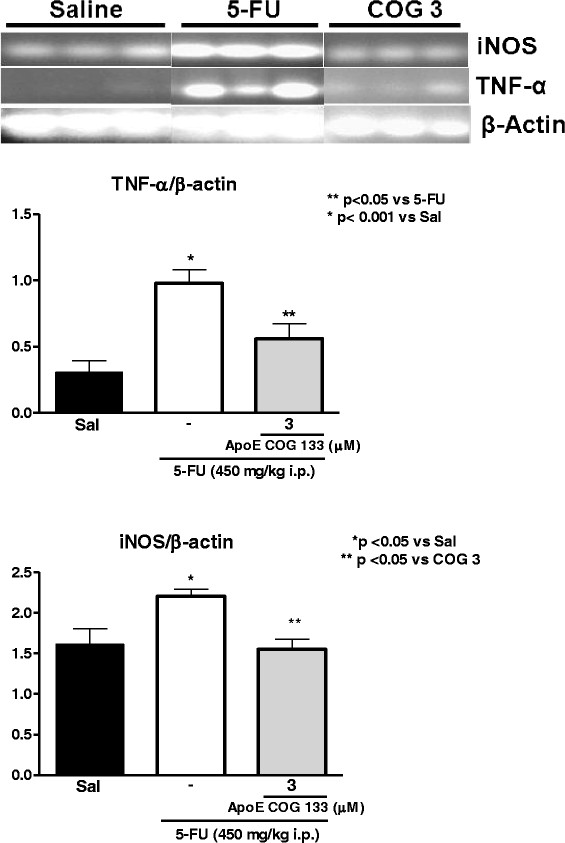
**Reverse-transcriptase analyses on tumor necrosis factor-alpha (TNF-α) and inducible nitric oxide synthase (iNOS) mRNA transcripts in the proximal intestine (N = 4 for each group).** Proximal intestinal segments were harvested from experimental mice on the fourth day. Densitometry results after normalized by β-actin are shown as mean ± SEM.

Our findings have shown increased intestinal TNF-α primary transcripts following 5-FU challenge, as compared to the controls (p <0.001), which was significantly reduced by the ApoE COG 133 peptide (3 μM). In addition, inducible nitric oxide synthase (iNOS) transcripts were increased in the proximal intestine after 5-FU challenge, as well. Likewise, the ApoE peptide treatment reduced iNOS mRNA expression (Figure 4).

#### Apoptosis detection by TUNEL assay

**Figure 5:**
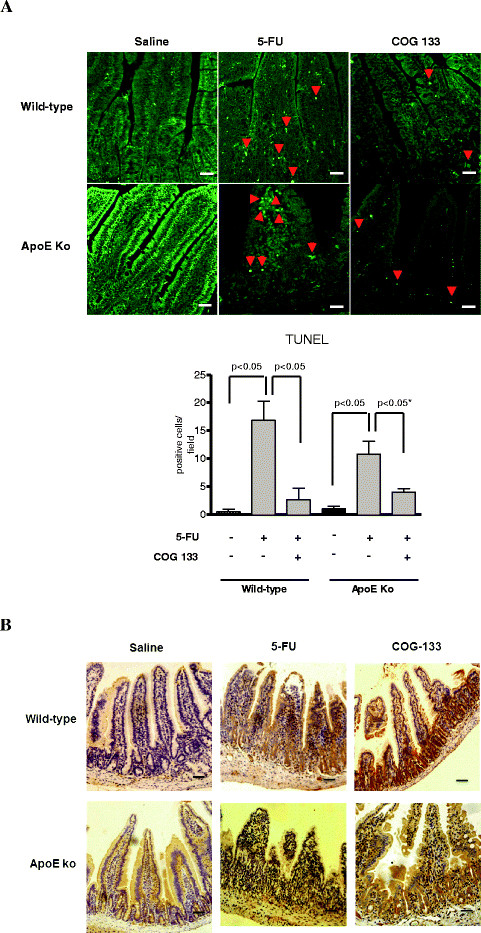
**A. Terminal deoxynucleotidyl transferase-mediated dUTP nick end-labeling (TUNEL) analysis of apoptosis in C57BL6J apoE knock-out and wild-type mice challenged by 5-FU-induced intestinal mucositis (450 mg/kg via i.p., single dose) and treated with the apoE mimetic peptide COG 133 (3 μM, via i.p., 12/12 h, vol. 10 μl/g) or 0.9% saline (i.p.).** Values represent mean ± SEM and were analyzed by one-way ANOVA and Bonferroni.. TUNEL-labeled cells (head arrows) were found increased 5-FU treated mice. Effect that was reversed by ApoE COG 133 peptide. Scale bars = 10 μm. B. NF-κB p50 representative immunohistochemistry of the proximal intestine tissue (X400) after four days of 5-FU-challenge. Note higher NF-κB immunlabeling in recovered crypts treated by ApoE COG 133 peptide. Scale bars, 10 μm.

Mice treated with 5-FU (450 mg/kg i.p.) showed a significant increase in Tunel-positive cells in the proximal intestine, an effect that was reverted by COG 133 administration (3 μM) in both wild-type and ApoE knock-out mice **(**Figure 5**A).** As expected, 5-FU treatment caused marked NF-κB immunolabeling of mucosa in wild-type mice. Interestingly, 5-FU-ApoE knock-out mice showed less intense NF-κB immunostaining as compared with their wild-type counterparts. In addition, the COG 133 treatment caused even higher expression of the NF-κB in the improved intestinal mucosa. NF-κB heavier labeling was associated with recovered crypt and villus epithelia (Figure 5B).

### In vitro studies

#### Proliferation assays

**Figure 6:**
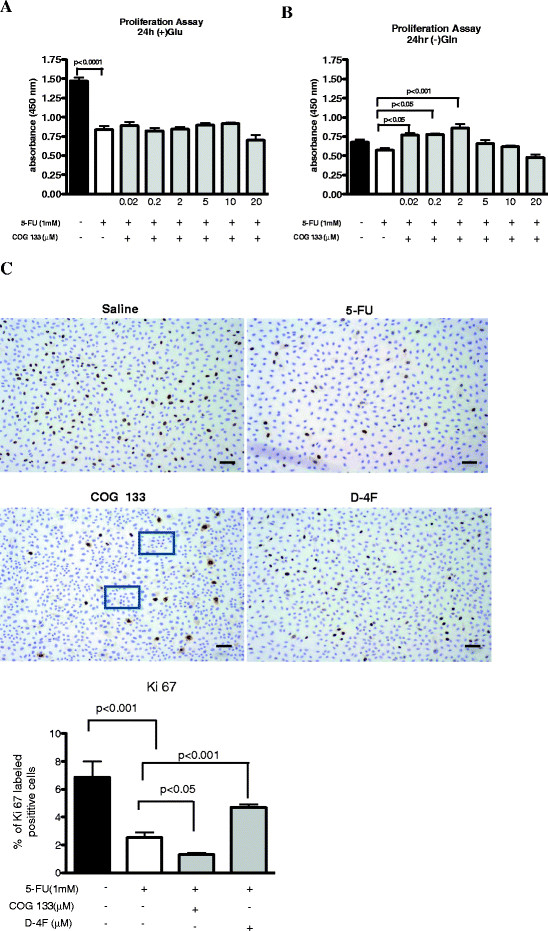
**Dose response of the apoE COG 133 mimetic peptide (0.02-20 μM) on IEC-6 cell proliferation 24 h after 5-FU challenge (5-FU media exposure for 1 h) in glutamine-free media (A) or standard media (B), detected by formazan absorbance using an ELISA microplate reader at 450 nm in 96-well seeded IEC-6 cells.** Values represent mean ± SEM and were analyzed by one-way ANOVA. (C) Ki67 immunolabeling of IEC-6 cells from chamber slides 24 h after 5-FU challenge (5-FU media exposure for 1 h) in glutamine-free media with either ApoE (COG 133) or ApoA-I (D-4F) peptides. Squares show areas of nested cells, suggesting a cloning cluster. Values represent mean% of Ki67-labeled cells in high magnified fields (X400) and were analyzed by one-way ANOVA.

IEC-6 monolayers challenged with 5-FU (1 mM for 1 h diluted in standard glutamine- containing media) showed reductions in cell numbers (lower mitochondrial activity, as measured by formazan detection) 24 h after 5-FU as compared to unexposed IEC-6 cells in glutamine enriched media (p < 0.001), suggesting that 5-FU increased glutamine uptake during 5-FU challenge. ApoE peptide treatment was not able to improve cell numbers in glutamine enriched media following 5-FU exposure in any of the doses tested. In fact, the highest dose of 20 μM caused significant cell proliferation impairments as compared to 5-FU controls (Figure 6A).

5-FU pre-treated IEC-6 monolayers showed reduced cell numbers, as compared with monolayers seeded in glutamine free-medium (p = 0.05). The ApoE peptide, at doses of 0.02, 0.2, and 2.0 μM, improved cell numbers in glutamine free media, suggesting a greater effect during a catabolic stress (Figure 6B). In order to measure mitotic activity we used Ki67 immunolabeling to further confirm the proliferative effect of COG 133. 5-FU-challenged monolayers showed lower Ki67 labeling (% of positive cells in higher magnified field). We found no improvement in Ki67 immunolabeling with either 2 or 20 μM COG 133. However we found increased numbers of cells showing and areas of clustering suggesting a proliferative nest. In order to evaluate the HDL receptor involvement, we have used D-4F, with significant improvements only at the highest dose (Figure 6C).

#### Wound healing model

**Figure 7:**
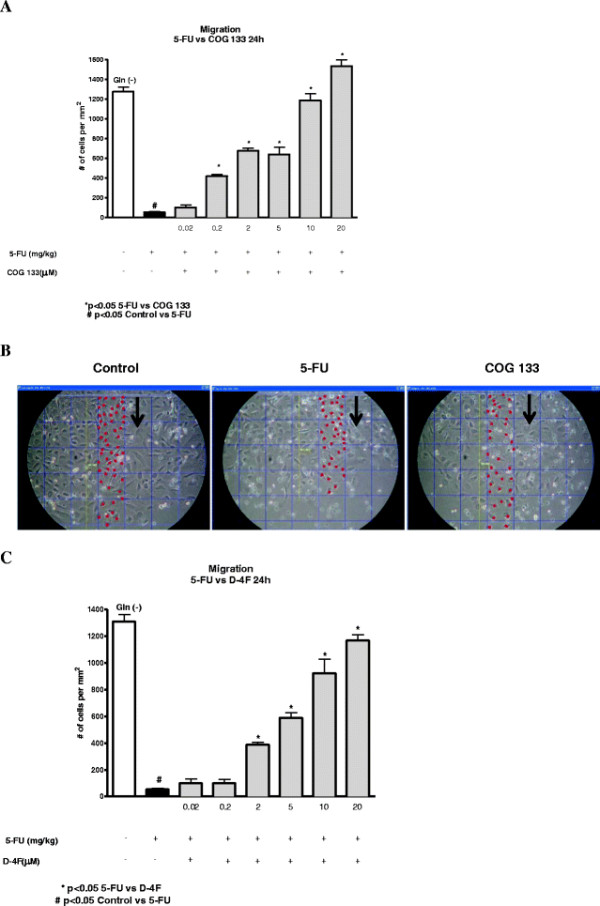
**A. ApoE mimetic (COG 133) effect on IEC-6 cell migration, 24 h after 5-FU challenge (5-FU media exposure, 1 mM, for 1 h).** Cell proliferation was analyzed under glutamine-free media using WST assay. **B**. ApoA-I (D-4F) peptide (0.02-20 μM) effect on IEC-6 cell migration, 24 h after 5-FU challenge (5-FU media exposure, 1 mM, for 1 h). Cell proliferation was analyzed under glutamine-free media using WST assay. Values represent mean ± SEM and were analyzed by one-way ANOVA.

5-FU (1 mM) markedly reduced IEC-6 cell migration in glutamine-free media, as compared to the unchallenged control (24 h post-challenge). COG 133 (0.2-20 μM) improved cell migration following 5-FU challenge (p = 0.0001), reaching the same migration level as controls (Figure 7A and B). When D-4F was tested under the same conditions, similar benefits were seen, however not at its lowest dose (Figure 7C).

#### Cell viability assays

**Figure 8:**
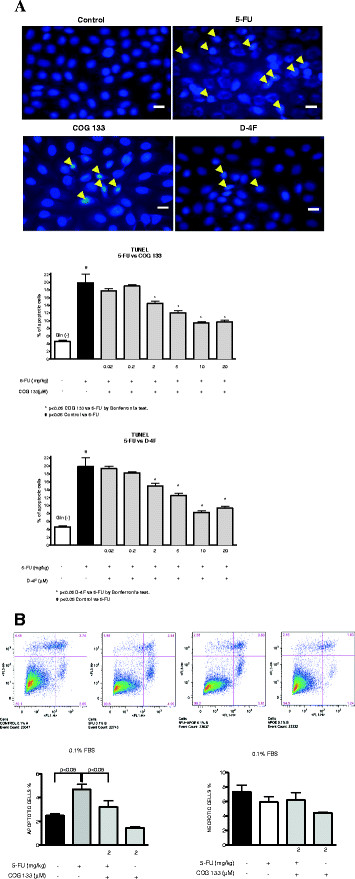
**A. Terminal deoxynucleotidyl transferase-mediated dUTP nick end-labeling (TUNEL) analysis of apoptosis in IEC-6 cells were analyzed under 5% fetal bovine serum and exposed to 5-FU, with either ApoE (COG 133) or ApoA-I (D-4F) peptide treatment.** Cells were stained using DeadEnd Fluorometric Tunel kit (head arrows). The Tunel System measures the fragmented DNA of apoptotic cells by catalytically incorporating fluorescein-12-dUTP at 3′-OH DNA ends using the enzyme TdT (Terminal deoxynucleotidyl Transferase). Data are plotted as mean TUNEL-labeled cells under high magnification (X400). Values represent mean ± SEM and were analyzed by one-way ANOVA. B. Cell necrosis and apoptosis were analyzed with flow cytometry in 1% fetal bovine serum and exposed to 5-FU with ApoE COG 133 peptide treatment. Cells were stained with FITC-conjugated annexin V and propidium iodide and analyzed by flow Calibur dual laser fluorescence-activated cell sorter (FACS). Results are shown as density plots with propidium iodide vs. annexin V-FITC. Bars on the graph represent the percentage of apoptotic cells have apoptotic and necrotic cells.

Using Tunel labeling, we found increased apoptotic figures (measured by % of apoptotic cells in total cell population at high magnification) after 5-FU challenge in glutamine free-media, effects that were ameliorated with either ApoE COG 133 or the D-4F peptides at the same doses (2–20 μM) (Figure 8**A)**.

We also tested the ApoE mimetic peptide under low bovine fetal serum concentrations with glutamine-free medium (1%), a stress condition that significantly reduces cell viability [17]. Triplicate flow cytometry analyses demonstrated, as expected, a significant increase in apoptosis in IEC-6 cells 24 h post-5-FU challenge. ApoE peptide treatment reduced cell apoptosis in glutamine-free media with low bovine fetal serum concentration (p < 0.05). However, when necrotic cells were considered for analysis, we found similar necrotic features in the glutamine-deprived IEC-6 controls and 5-FU-challenged cells (p > 0.05). In addition, ApoE peptide treatment did not reduce necrotic cells compared to both 5-FU-exposed and glutamine-deprived IEC-6 cells (Figure 8B).

## Discussion

5-FU is one of the most prescribed drugs in clinical oncology for colo-rectal cancer patients [18,19], however side effects are often reported, leading to compliance failure. The intestinal barrier function may suffer from 5-FU cytotoxic insults in various ways, including mucosal inflammatory reactions, intestinal epithelial barrier disruption, and bacterial translocation [20].

The data described here are in accordance with previous work from Soares *et al*., who followed 5-FU-treated rats (1°, 3°, 5°, 15° and 30° day) and showed marked leukopenia, MPO activity, inflammatory cell infiltration, villus shortening, and crypt destruction in the duodenal lamina propria on the third day post-5-FU challenge [5].

Interestingly, we found a marked crypt mitotic arrest in 5-FU treated mice, which was ameliorated by COG 133 peptide only in wild-type but not ApoE knock-out mice, as shown by our findings of crypt mitotic index. This may be caused by a disrupted growth factor signaling, since we had shown impaired intestinal adaptations during catch-up growth following re-feeding in severe undernourished ApoE knock-out mice, with poor intestinal IGF-1 expression [21].

Our data suggest that ApoE mimetic peptide may modulate inflammation by increasing NF-κB expression in recovered epithelia from duodenal crypts, therefore may be also activating the NF-κB anti-inflammatory pathway. NF-κB signaling has been found to improve epithelial migration after intestinal injury [22], and has been shown to be associated with cyclin 1 expression [23], both factors that could improve crypt recovery from 5-FU epithelial damage. Although, COG 133 peptide could also enhance intestinal mucosal repair in ApoE knock-out mice, the NF-κB immunolabeling was less increased than in the wild-type mice following ApoE-peptide treatment. Further studies are warranted to examine this effect in more depth, especially addressing growth factors involved in mucosal restitution and their regulation by ApoE peptides.

Recent studies in rats demonstrated that 5-FU administration (150 mg/kg i.p.) significantly increased MPO activity (275%) in the jejunum, as compared with unchallenged controls [24]. In addition, increased concentrations of TNF-α and IL-1β with involvement of platelet-activating factor were found in the rat duodenum [25]. In order to assess inflammatory responses to 5-FU challenge, we measured ileal cytokines and MPO levels. Our findings also showed marked increases in intestinal MPO activity on the 3rd day after 5-FU challenge (450 mg/kg i.p), effects that were abrogated in mice treated with 3 μM COG133 peptide, suggesting a reduction of the MPO-producing inflammatory cell infiltrates by the peptide.

In our study, we detected increased levels of IL-1β and TNF-α and their transcripts (and reduced IL-10 levels) and increased expression of the inflammatory mediator iNOS mRNA in the proximal small intestine among 5-FU challenged Swiss mice, findings that were partially abrogated by ApoE mimetic peptide treatment, supporting an anti-inflammatory role of the ApoE mimetic peptide in the 5-FU-driven intestinal mucositis. This finding of ApoE modulatory action on pro-inflammatory cytokines is reinforced by reports of a pro-inflammatory state in mice lacking the APOE gene [26] and poor survival of these animals in models of sepsis [27]. Studies by Lynch and colleagues have demonstrated an anti-inflammatory effect of the ApoE peptide, COG 133, when administered intra-peritoneally to LPS-challenged (i.p.) mice, leading to significant reductions in serum TNF-α after 1 and 3 hours. Such anti-inflammatory effect was also found in the brain tissue of these mice, with reductions in the TNF-α mRNA expression 3 hours after LPS injection [8]. Furthermore, an anti-LPS role is also postulated in studies using human recombinant ApoE. ApoE- enriched emulsion can shift bacterial LPS from liver Kupffer cells to hepatocytes, therefore reducing LPS-derived endotoxemia (binds at ratio of 1:2 LPS molecules) [28,29]. These findings suggest that the benefit of ApoE mimetic peptide we observed may have been through blocking effects of bacterial (or LPS) translocation following 5-FU-induced intestinal barrier leakage.

Regarding mediators that might be altered by ApoE mimetic peptides, TNF-α and IL-1β are important pro-inflammatory cytokines activated by NF-κB pathway, which is up-regulated during the pathogenesis of 5-FU-induced mucositis [30][31]. Interestingly, the ApoE COG-112 mimetic peptide was found to inhibit NF-κB signaling and downstream pro-inflammatory cytokines in *Citrobacter rodentium*-induced colitis in mice [32,33]. In this model, authors also found reductions in tissue neutrophil infiltration, corroborating our findings of lower MPO levels (as a neutrophil marker) in intestine with ApoE peptide treatment. In addition, IL-1 receptor antagonist was found to reduce intestinal toxicity of 5-FU in mice, suggesting that IL-1 peptides are key players in the intestinal inflammatory response. Our findings with NF-κB suggest that although this signaling pathway is involved in the inflammatory response, it may be important for crypt recovery following 5-FU-induced epithelial damage.

Our findings that ApoE deficient mice show higher TUNEL-labeled cells, more inflammation and more blunted villi than wild-type controls, reinforce the role of ApoE in protecting the intestinal mucosa against 5-FU-induce intestinal mucositis.

In our in vitro studies, however we only find improvements in the number of Ki67-labeled IEC-6 cells with ApoA-I peptide using a high treatment dose (20 μM). However, increased cell numbers were found with COG 133, suggesting that the peptide increased cell proliferation before Ki67-stained mitoses occurred. On the other hand, we found beneficial effects of COG 133 in lower doses using the WST assay, a method that measures the mithocondrial-formazan product seen in viable cells. These differences may relate to the time periods assessed, still under blunting mitotic effects following 5-FU treatment [34]. In addition, WST measurements show stronger proliferative effects of COG 133 in glutamine free media (rather than standard media with glutamine) in IEC-6 cells following 5-FU challenge, suggesting that the peptide has a potential biological effect under catabolic disease conditions when glutamine is more likely deprived.

In order to compare the ApoE mimetic peptide effect on cell migration and apoptosis, we also conducted *in vitro* experiments with ApoA-I (D-4F), which we found causing similar benefits to those seen with the ApoE peptide. Thus, both may affect a common downstream pathway. However, both COG 133 and the D-4F peptides are amphipathic alpha helices. Thus, the other possibility is that they can cross plasma membranes and enter the cell cytoplasm and have similar effects in a receptor-independent fashion.

Noteworthy is that the intestine is a major site of action of this peptide [35]. Recently, a novel anti-inflammatory mechanism of ApoE-mimetic peptides was identified, with inhibition of SET, a protein capable of increasing endogenous PP2A phosphatase activity, which reduces levels of phosphorilated kinase signaling and inflammatory cytokines [36]. SET is also involved in neuronal apoptosis due to amyloid precursors [37]. If the same mechanism is operating in the IEC-6 cell apoptotic machinery, the inhibition of SET by ApoE mimetic peptide would be anti-apoptotic, as seen in our flow cytometry studies after 5-FU challenge. Increased TNF-α-induced apoptosis would be also counterbalanced by the anti-inflammatory actions of the peptide, possibly mediated by a fine regulation of serine-threonine kinase 1 (RIP-1) ubiquitinization and caspase signaling [38]. At this point, the exact mechanism of the anti-apoptotic property of the ApoE COG 133 remains unclear.

In contrast to anti-proliferative findings of COG 112 and COG 133 peptides in lymphocyte cultures after myelin oligodendrocyte glycoprotein (MOG) exposure in a murine model of multiple sclerosis [11], our *in vitro* data in crypt IEC-6 cells in addition to the observation of improved crypt histology in treated mice suggest a beneficial role of the peptide in improving cell viability and crypt cell renewal following 5-FU challenge

Previous studies have shown that mucositis is not only a manifestation of tissue change, but also with systemic effects, with elevation of proinflammatory cytokines in serum, such as TNF-α, IL-1β, and IL-6 of 5-FU-treated patients, in association with the chemotherapy toxicity without any improvements in its anti-tumor activity [39].

Colton *et al*. demonstrated an APOE allelic-distinct effect on microglial nitric oxide production in the transgenic mouse brain [40,41]. A significant increase in NO production was found in isolated macrophages from human APOE4 targeted transgenic mice by activation of cationic amino acid transporters (CAT) with up-regulated innate immune system [41]. Therefore, a possible downregulation of CAT-1 transporters during a pro-inflammatory state of the intestinal mucositis would contribute to lower NO generation and thus milder inflammatory cytokine production.

Studies from Hoane *et al*. in models of brain injury have demonstrated improvements in cognitive tests following ApoE peptide treatment in rats. There was a dose-dependent peptide (COG 1410) effect, with the best dose found of 0.8 μg/kg, in studies showing recovery following traumatic brain injury [42]. While the mechanism of this benefit is unclear, it may involve an anti-inflammatory effect like that which we have seen.

## Conclusion

In summary, our in vivo and in vitro findings suggest a potential protective role of the novel COG 133 peptide by improving intestinal cell viability and migration, and potentially by counteracting the exacerbated inflammatory responses following 5-FU-induced intestinal mucositis.

Although our findings suggest an anti-inflammatory action of the COG133 peptide with the involvement of the LDL pathway, we cannot rule out nonspecific effect via a non-LDL route. Caution is also needed regarding the applicability of our data to the clinical setting, since our study did not evaluate anti-tumor effects of the COG133 peptide. Further studies are needed to corroborate our findings in order to support future clinical trials to ameliorate the side effects of chemo/radiotherapy-associated mucositis.

## Competing interests

All authors with the exception of M. P. Vitek declare no competing interests. Dr. Vitek is an employee of Duke University Medical Center and of Cognosci, Inc., of which he is the founder.

## Author’s contributions

OGRA and CVA conducted in vivo studies with Swiss and C57BL6J mice and data analyses. RACO, BCO, and CAW contributed to the IEC-6 in vitro studies. DVTW and RCPLJ conducted the immune assays. MPV contributed with the ApoE mimetic peptide experiments. TBC, SZ, and HBML contributed with TUNEL and Ki67 studies. RAR, AAML, RLG and RBO were responsible for the study design, statistical analyses, and research laboratory coordination. All authors read and approved the final manuscript.

## Pre-publication history

The pre-publication history for this paper can be accessed here:

http://www.biomedcentral.com/1471-230X/12/35/prepub
